# Translational insights into pain mechanisms and balance impairments in aging: a cross-sectional study

**DOI:** 10.3389/fnagi.2025.1656854

**Published:** 2025-10-20

**Authors:** Mastour Saeed Alshahrani, Ravi Shankar Reddy

**Affiliations:** Program of Physical Therapy, Department of Medical Rehabilitation Sciences, College of Applied Medical Sciences, King Khalid University, Abha, Saudi Arabia

**Keywords:** postural stability, pain intensity, pressure pain threshold, pain catastrophizing, chronic pain

## Abstract

**Objectives:**

This study aimed to investigate the association between pressure pain threshold (PPT), pain intensity, pain catastrophizing, and postural stability and to assess the impact of pain chronicity and duration on functional reach and posturographic outcomes in community-dwelling older adults.

**Methods:**

A cross-sectional study was conducted involving 136 older adults (mean age = 74.23 ± 6.52 years). Pain mechanisms were assessed using an algometer (PPT), the Numeric Pain Rating Scale (NPRS), and the Pain Catastrophizing Scale (PCS). Balance was evaluated via force plate posturography (sway metrics) and the Functional Reach Test (FRT). Covariates included Mini-Cog and Geriatric Depression Scale scores. Data were analyzed using Pearson correlations, multiple linear regression, and ANCOVA.

**Results:**

Sway velocity was significantly predicted by PPT (*B* = 0.47, *p* < 0.001), NPRS (*B* = −0.36, *p* < 0.001), PCS (*B* = −0.29, *p* = 0.001), Mini-Cog (*B* = 0.33, *p* = 0.003), and GDS (*B* = −0.18, *p* = 0.011), explaining 48% of the variance (R^2^ = 0.48, F(5,130) = 24.15, *p* < 0.001). Chronic pain was associated with reduced FRT performance (*F* = 9.45, *p* = 0.003), and longer pain duration predicted greater sway area (*B* = 0.014 ± 0.004, *p* = 0.001).

**Conclusion:**

Both sensory and cognitive-affective dimensions of pain, along with pain chronicity, are independently associated with postural stability impairments in older adults. These findings support the integration of multidimensional pain assessments in clinical balance evaluations.

## 1 Introduction

Maintaining postural stability is fundamental to functional independence and quality of life in older adults (Chiu et al., 2021). With advancing age, physiological changes in the neuromuscular and sensory systems compromise balance control, contributing to increased fall risk and reduced mobility (Rodrigues et al., 2022). Falls are a leading cause of injury, disability, and mortality in the elderly population, with significant personal and healthcare-related consequences (Van Deynse et al., 2022). While age-related declines in balance and strength have been widely studied, emerging evidence suggests that the role of pain–particularly chronic musculoskeletal pain–is an underappreciated yet influential factor in postural regulation (Fyfe et al., 2022). Given the high prevalence of persistent pain among older adults, understanding how pain mechanisms interact with balance control systems is essential for developing targeted interventions to prevent falls (Xing et al., 2023).

Pain is not solely a sensory experience but is also modulated by cognitive and affective processes (Khera and Rangasamy, 2021). Constructs such as pain catastrophizing and perceived pain intensity have been shown to influence movement behavior and physical function. Pain-related fear, maladaptive beliefs, and attentional biases may alter motor planning and postural adjustments, potentially exacerbating balance deficits (Chau et al., 2023). Furthermore, chronic pain has been linked to alterations in proprioceptive processing and neuromuscular control, which are essential for maintaining upright posture (Alvani et al., 2021). Despite these theoretical underpinnings, the integration of pain sensitivity, cognitive-emotional pain responses, and chronicity into models of balance dysfunction in aging remains limited (Van Wesemael et al., 2024). Existing literature continues to highlight this gap, with few comprehensive frameworks incorporating sensory and affective pain dimensions in the context of postural control (Van Wesemael et al., 2024; Viseux et al., 2022). Quantitative sensory testing methods, such as pressure pain threshold (PPT) assessment, provide objective measures of nociceptive sensitivity. At the same time, validated scales like the Numeric Pain Rating Scale (NPRS) and Pain Catastrophizing Scale (PCS) offer insights into subjective pain experiences (Arant et al., 2022). The combined influence of these pain dimensions on postural stability is not well established, particularly in community-dwelling older adults (Sarvari et al., 2024).

Previous studies investigating pain and balance have often focused on isolated variables or specific clinical populations, without simultaneously accounting for the multidimensional nature of pain and its chronicity (Sarvari et al., 2024; Viseux et al., 2022). This limitation has been echoed in recent empirical studies examining pain-related factors in older adults with low back pain and osteoarthritis, where multidimensional assessments were often absent or inconsistently applied. Multiple investigations have emphasized the necessity of evaluating both nociceptive and cognitive-affective components when examining postural outcomes in older adults, highlighting the complex interaction between pain perception and balance control (Ahmadi et al., 2025; Gramani-Say et al., 2025). Few studies have incorporated both sensory and psychological pain metrics in relation to objective posturographic measures and functional balance tests (Alshahrani and Reddy, 2024). Alshahrani et al. (2025), Alshahrani and Reddy (2024) and Knox et al. (2021) have demonstrated that psychological factors such as kinesiophobia and pain-related beliefs, alongside physical measures of pain, significantly contribute to balance impairments and fall risk in older adults with musculoskeletal conditions. Moreover, the influence of covariates such as cognitive function and depressive symptoms–known to impact both pain perception and balance–has not been consistently controlled (Alfaya et al., 2023). Peterson et al. (2023) underscore the moderating roles of cognitive status and emotional well-being on physical performance, suggesting these variables are essential when modeling pain-related balance dysfunction. This methodological gap limits the clinical applicability of current findings and impedes the development of comprehensive fall-risk assessment protocols in geriatric care. A more precise understanding of how discrete pain mechanisms and chronic pain exposure impact postural control could inform more effective, personalized intervention strategies.

Therefore, the present study aimed to examine the association between pressure pain threshold, pain intensity, and pain catastrophizing with postural stability in older adults using both functional and posturographic outcome measures. In addition, it investigated the relationship between pain chronicity and characteristics of balance performance, accounting for cognitive and affective factors. It was hypothesized that lower PPT, higher pain intensity, and greater catastrophizing would be significantly associated with poorer postural stability and that individuals with chronic pain would demonstrate greater balance impairments compared to those with acute or subacute pain. By integrating multidimensional pain and balance assessments, this study seeks to provide a comprehensive analysis of the mechanisms linking pain and postural control in aging populations.

## 2 Materials and methods

### 2.1 Design

This cross-sectional observational study was conducted between June 2023 and April 2024 at the Physical Therapy Clinic at DMRS, Kingdom of Saudi Arabia. The study protocol was reviewed and approved by the institutional ethics committee [approval number: (CEM#2023-287)], and all procedures adhered strictly to the principles outlined in the Declaration of Helsinki. All participants provided written informed consent, and confidentiality of personal data was maintained in accordance with institutional and international standards. Safety during testing was ensured by having trained physiotherapists supervise all procedures, with protocols in place to minimize fall risk and immediately address any adverse events.

### 2.2 Sample size calculation

The sample size for this study was determined *a priori* using G*Power statistical software to ensure adequate power for detecting significant associations between pain-related variables and postural stability outcomes in older adults. A linear multiple regression model was selected with five predictor variables: PPT, pain intensity (NPRS), pain catastrophizing (PCS), Mini-Cog score, and Geriatric Depression Scale (GDS) score. Based on effect sizes reported in previous cross-sectional studies evaluating pain and balance relationships in older adults, a medium effect size (f^2^ = 0.15) was assumed. The alpha level (α) was set at 0.05, with a desired statistical power of 0.80 (1−β), which are standard parameters for behavioral and clinical research. Using these inputs, the minimum required sample size was calculated to be 92 participants. To account for potential dropouts and missing data and to increase the precision of estimates, the sample size was inflated by 30%, resulting in a final target sample size of 136 participants.

### 2.3 Participants

Participants were community-dwelling older adults who attended the Physical Therapy Clinic at the Department of Rehabilitation Medicine for musculoskeletal consultations between June 2023 and April 2024. At the time of recruitment, participants had not yet initiated physical therapy treatment; all assessments were conducted during or immediately following their initial consultation visit, prior to the commencement of any therapeutic intervention. Therefore, no participant was undergoing physical rehabilitation during data collection, and this variable was not included as a covariate in the analysis. Individuals were recruited consecutively using convenience sampling based on their willingness and eligibility to participate. The diagnostic criteria for inclusion required the presence of musculoskeletal pain, either acute (less than 3 months) or chronic (3 months or more) (El-Tallawy et al., 2021), as determined through structured clinical interviews and self-reported pain duration. Participants were screened by a licensed physical therapist to confirm the musculoskeletal origin of pain based on clinical examination and history.

Eligible participants were aged 60 years or older and able to ambulate independently with or without assistive devices. Additional inclusion criteria included the ability to comprehend study instructions and complete questionnaires, a Mini-Cog score of ≥3 indicating preserved cognitive function. Exclusion criteria comprised a history of neurological conditions affecting balance, severe visual or vestibular impairments, diagnosed dementia, recent orthopedic surgery (within the past 6 months), or any acute medical condition contraindicating participation in balance testing. Participants who met the inclusion criteria and provided written informed consent were enrolled and underwent baseline assessment as part of the study protocol.

### 2.4 Postural control assessment

**FIGURE 1:**
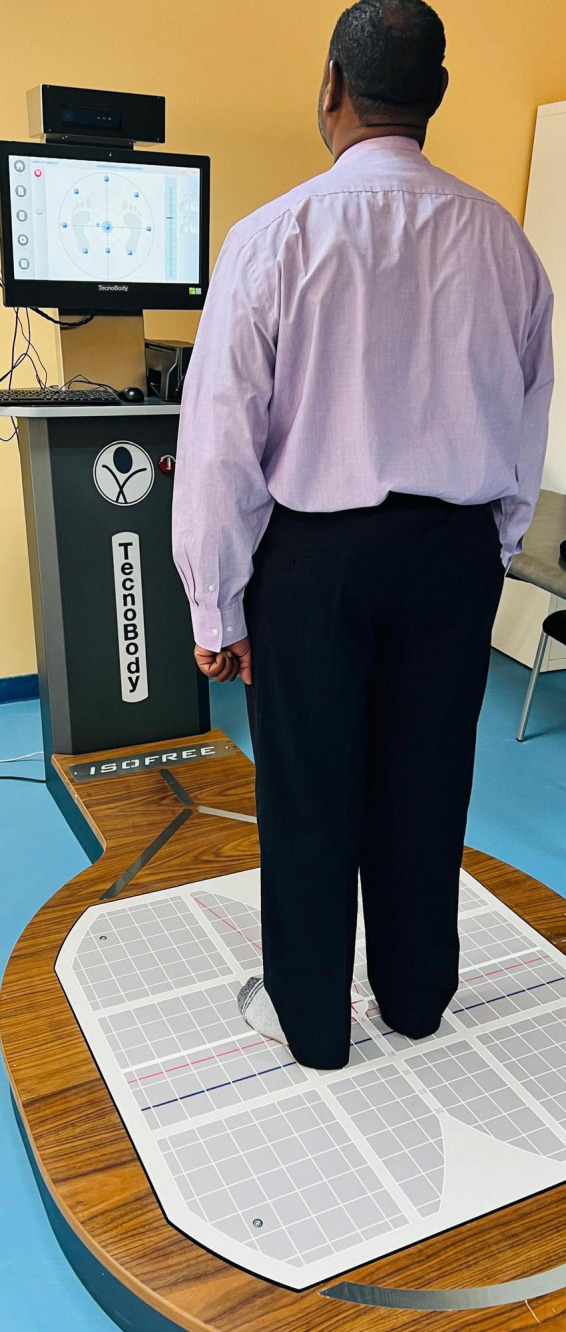
Static posturography assessment using the tecnobody isofree system.

The primary outcome of postural stability was assessed using static posturography performed on a computerized force plate system, a validated tool widely used in clinical and research settings (Janc et al., 2021). Participants were assessed in a standardized testing environment with ambient lighting and minimal external distractions. Prior to the test, each participant was given a clear explanation and demonstration of the procedures, and a familiarization trial was conducted to minimize variability due to apprehension or misunderstanding. Participants were instructed to stand barefoot in an upright position on the force platform with their feet positioned shoulder-width apart, aligned symmetrically using pre-marked foot placements on the platform to ensure consistency across trials (Figure 1). Arms were kept relaxed at the sides, and participants were instructed to keep their gaze fixed on a visual target at eye level, located 2 m ahead on the wall, to minimize head movements and visual tracking interference. All trials were conducted with eyes open, and participants were advised to remain as still as possible throughout the data acquisition period. Each posturographic recording lasted for 30 s, during which the system captured center-of-pressure (CoP) movements at a sampling rate of 100 Hz. The primary sway variables extracted were sway velocity (cm/s), sway area (cm^2^), mediolateral sway (cm), and anteroposterior sway (cm). Three trials were performed with a 1-min rest interval between trials to prevent fatigue. The average of the three trials was used for analysis to improve the reliability of the measurements.

### 2.5 Functional balance assessment

Functional balance was concurrently assessed using the Functional Reach Test (FRT) (Soke et al., 2022), a simple and valid measure of anterior-posterior dynamic stability. Participants were instructed to stand next to a wall-mounted measuring device with their dominant arm extended at 90 degrees of shoulder flexion, parallel to the floor. Without moving their feet, they were asked to reach forward as far as possible without losing balance or taking a step. The distance reached, measured in centimeters, was recorded from the starting position (third metacarpal head) to the furthest reach. Three consecutive trials were conducted, and the mean value was used in the final analysis. All assessments were conducted by trained physiotherapists following a standardized testing script and protocol to ensure consistency and reduce inter-rater variability.

### 2.6 Pain sensitivity and pain intensity assessment

**FIGURE 2:**
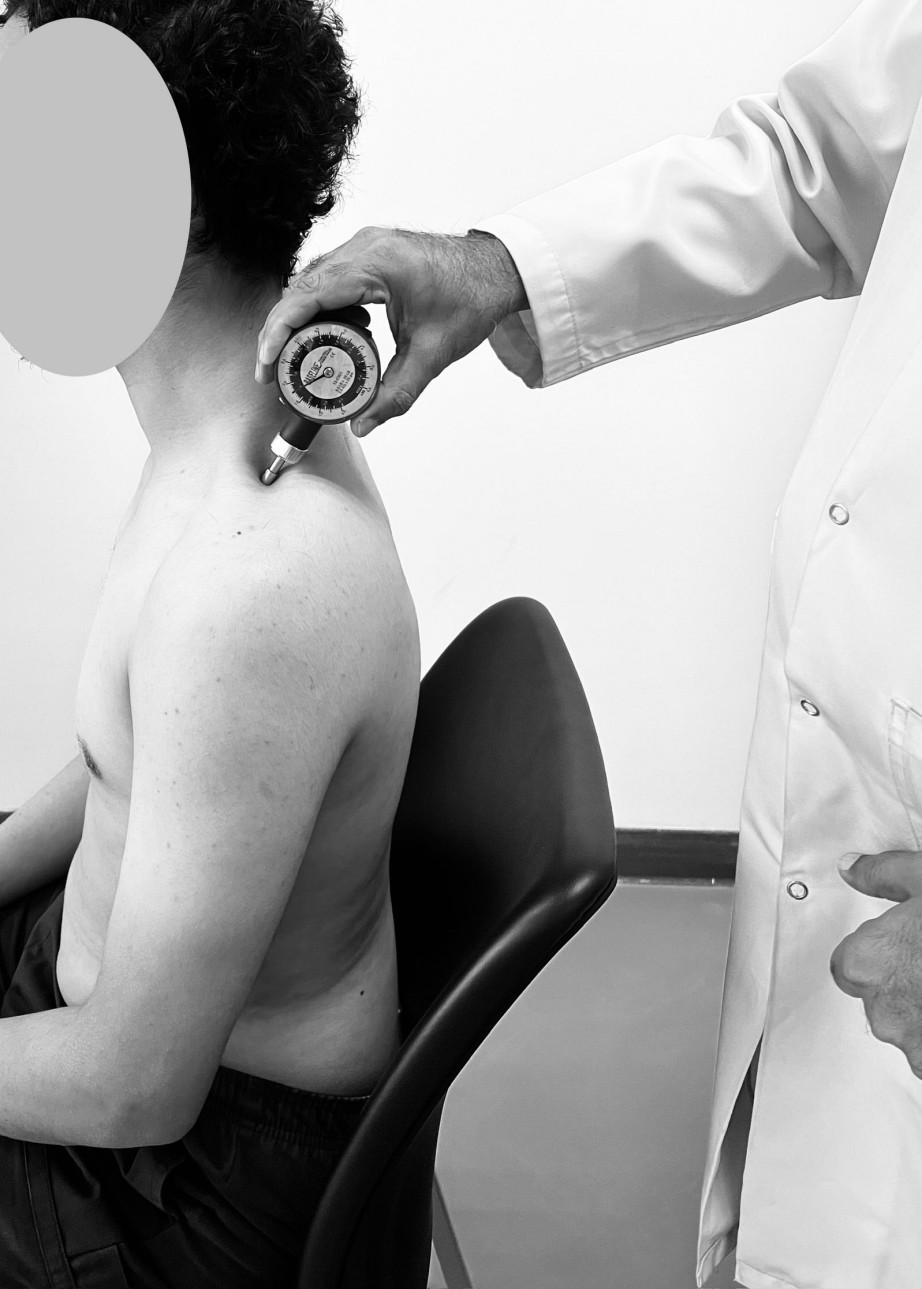
Pressure pain threshold (PPT) assessment using a digital algometer.

Pain sensitivity was operationalized using the PPT, an objective and quantifiable measure of mechanical nociceptive sensitivity. PPT was assessed using a digital pressure algometer (Model: Wagner FPX, Wagner Instruments, Greenwich, CT, USA), a handheld device equipped with a 1 cm^2^ rubber-tipped probe capable of applying controlled and measurable pressure. Participants were seated comfortably with adequate support, and the testing site was exposed while maintaining privacy. The anatomical site chosen for PPT testing was the midpoint of the upper trapezius muscle on the dominant side, located halfway between the acromion and the spinous process of C7. This site was marked with a dermatological pencil to ensure consistent probe placement across trials. The algometer was held perpendicular to the skin surface, and pressure was increased steadily at a rate of approximately 1 kg/cm^2^ per second (Figure 2). Participants were instructed to verbally indicate the exact moment the pressure sensation changed to slight pain; this point was recorded as the pressure pain threshold. To improve measurement reliability and reduce random error, three consecutive readings were obtained with a rest interval of 30 s between trials. The mean of the three values (expressed in kg/cm^2^) was used in subsequent statistical analyses. All measurements were conducted by trained examiners with prior experience in quantitative sensory testing to ensure procedural consistency and minimize inter-rater variability. Pain intensity was measured using the 11-point Numeric Pain Rating Scale (NPRS), with scores ranging from 0 (no pain) to 10 (worst imaginable pain), based on the average pain intensity reported in the past week. Pain chronicity was classified based on self-reported duration of pain. Pain lasting less than 3 months was categorized as acute/subacute, whereas pain persisting for 3 months or more was classified as chronic. Pain duration was recorded in months through structured interviews during the initial screening.

### 2.7 Covariates

Two covariates were included to control for potential confounding effects: cognitive function and depressive symptoms. Cognitive function was assessed using the Mini-Cog, which includes a three-item recall test for memory and a clock-drawing test for executive function. Scores range from 0 to 5, with scores ≥3 indicating intact cognition. Depressive symptoms were measured using the 15-item Geriatric Depression Scale (GDS) (Kim et al., 2021), a validated screening tool designed to assess depressive symptomatology in older adults. Participants responded to yes/no questions reflecting mood, motivation, and life satisfaction over the past week. Scores range from 0 to 15, with higher scores indicating greater depressive symptoms. A cutoff score of >5 suggests the presence of mild depressive symptoms, while scores ≥10 are indicative of more severe depression (Brañez-Condorena et al., 2021).

### 2.8 Data analysis

Prior to analysis, data were screened for missing values and outliers, and the assumption of normality was confirmed using Shapiro–Wilk tests. Pearson correlation coefficients were calculated to examine bivariate associations between pain-related variables (PPT, NPRS, PCS, and pain duration) and postural stability outcomes (sway velocity, sway area, and FRT). Multiple linear regression analysis was conducted to determine the independent predictors of sway velocity, adjusting for age, cognitive function (Mini-Cog), and depressive symptoms (GDS). Additionally, an ANCOVA was used to compare functional reach performance between chronic and acute pain groups, controlling for age and GDS scores. A linear regression model was also used to assess the relationship between pain duration and sway area, adjusting for age and GDS scores only, as Mini-Cog was not significantly associated with sway area in bivariate analysis.

## 3 Results

Participants in this study were predominantly female (61.76%), with a mean age of 74.23 years, representing a typical community-dwelling older adult cohort (Table 1). The average body mass index indicated a slightly overweight population, and the mean pain duration exceeded 3 years, supporting the chronic nature of pain in this sample. Notably, knee pain was the most commonly reported location. Cognitive and mood assessments revealed generally preserved cognition and low depressive symptomatology. The average pressure pain threshold and pain intensity scores suggest moderate nociceptive sensitivity and pain experience, while pain catastrophizing scores were consistent with mild to moderate cognitive-affective distress. Balance assessments demonstrated reduced functional reach and elevated sway parameters, indicative of compromised postural stability in this aging population. Additional descriptive characteristics are provided in Supplementary Table 1.

**TABLE 1:** Demographic and clinical characteristics of participants (n = 136).

Variable	Mean ± SD or *n* (%)
Age (years)	74.23 ± 6.52
Sex (male/female)	52 (38.24%)/84 (61.76%)
BMI (kg/m^2^)	26.41 ± 3.92
Pain duration (months)	38.75 ± 21.58
Pain location (back/knee/generalized)	45 (33.09%)/61 (44.85%)/30 (22.06%)
Cognitive status (mini-cog score)	3.47 ± 1.12
Mood (GDS score)	2.13 ± 1.05
Comorbidities (diabetes/arthritis/none)	34 (25.00%)/49 (36.03%)/53 (38.97%)
Pressure pain threshold (PPT, kg/cm^2^)	3.82 ± 1.04
Pain intensity (NPRS, 0–10)	5.26 ± 1.93
Pain catastrophizing scale (PCS)	21.34 ± 9.87
Functional reach test (FRT, cm)	22.45 ± 6.21
Sway velocity (cm/s)	3.42 ± 1.01
Sway area (cm^2^)	2.15 ± 0.88
Mediolateral sway (cm)	1.46 ± 0.56
Anteroposterior sway (cm)	2.62 ± 0.71

BMI, body mass index; GDS, Geriatric Depression Scale; Mini-Cog, mini-cognitive assessment instrument; PPT, pressure pain threshold; FRT, functional reach test; SD, standard deviation.

**FIGURE 3:**
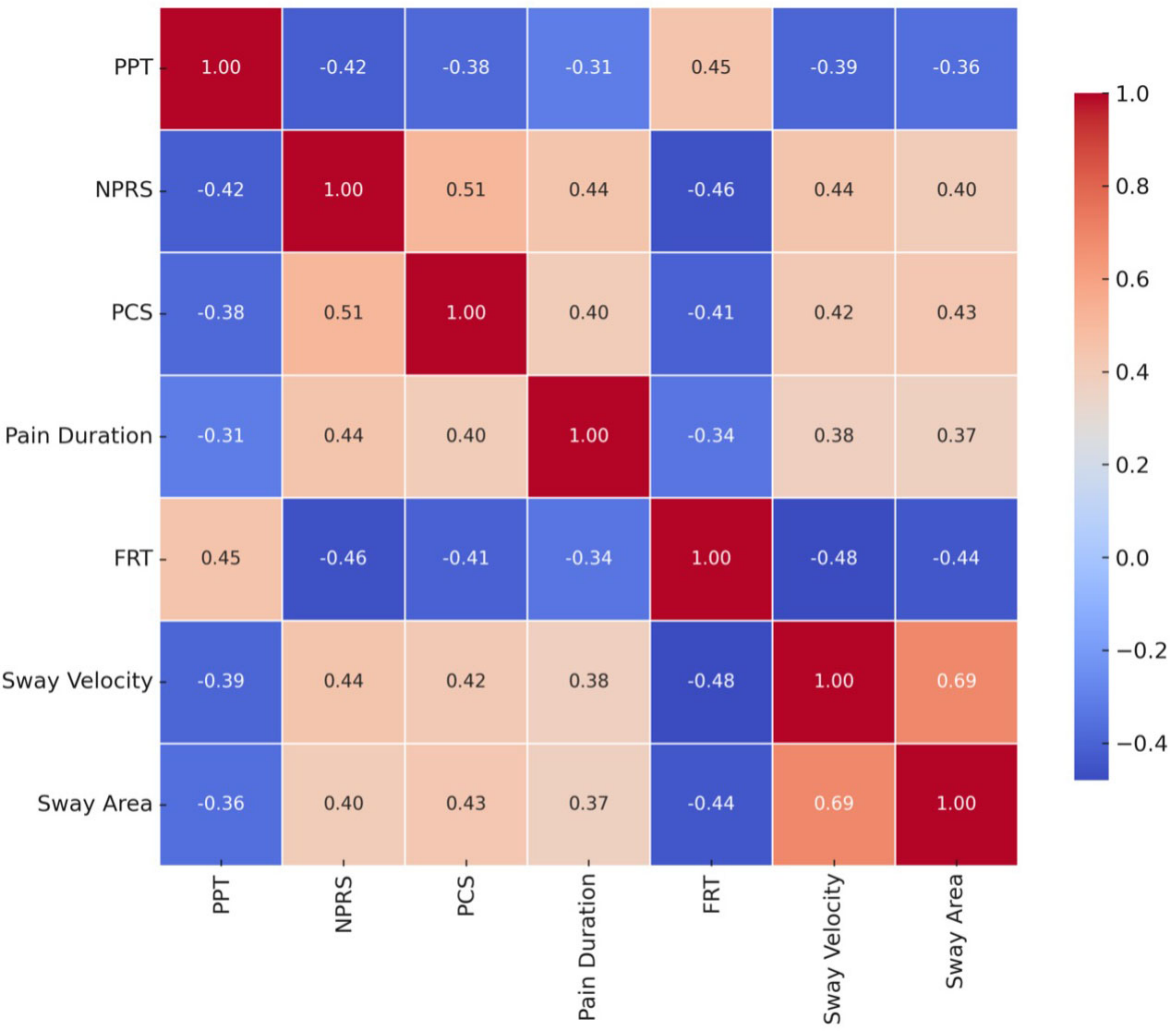
Correlation matrix among pain mechanisms and postural control outcomes in older adults.

Significant correlations were found between pain-related variables and postural control measures, indicating meaningful interactions across sensory, cognitive-affective, and functional domains in older adults (Figure 3). Lower pressure pain threshold (PPT) was moderately associated with higher pain intensity (*r* = −0.42, *p* = 0.003), greater pain catastrophizing (*r* = −0.38, *p* = 0.007), and better functional reach performance (*r* = 0.45, *p* = 0.002). Pain intensity (NPRS) showed strong positive correlation with catastrophizing (PCS) (*r* = 0.51, *p* < 0.001), and was negatively associated with functional reach (*r* = −0.46, *p* = 0.001), while positively related to sway velocity (*r* = 0.44, *p* = 0.002) and sway area (*r* = 0.40, *p* = 0.005). Pain duration was positively correlated with PCS (*r* = 0.40, *p* = 0.004), sway velocity (*r* = 0.38, *p* = 0.006), and sway area (*r* = 0.37, *p* = 0.008). Sway velocity demonstrated the strongest associations overall, correlating with sway area (*r* = 0.69, *p* < 0.001), PCS (*r* = 0.42, *p* = 0.003), and NPRS (*r* = 0.44, *p* = 0.002), supporting its role as a key indicator of postural instability in this cohort.

The multiple linear regression model significantly predicted sway velocity, explaining 48% of the variance in postural stability among older adults (R^2^ = 0.48, F(5, 130) = 24.15, *p* < 0.001) (Table 2). Pressure pain threshold (*B* = 0.47, 95% CI [0.24, 0.70], *p* < 0.001) was the strongest positive predictor, indicating that higher mechanical sensitivity was associated with greater postural instability. Conversely, higher pain intensity (NPRS; *B* = −0.36, 95% CI [−0.54, −0.18], *p* < 0.001) and greater pain catastrophizing (PCS; *B* = −0.29, 95% CI [−0.45, −0.13], *p* = 0.001) were both independently associated with reduced sway velocity, suggesting a complex interaction between sensory and cognitive-emotional pain processing. Additionally, cognitive status (Mini-Cog; *B* = 0.33, *p* = 0.003) positively contributed to sway control, while depressive symptoms (GDS; *B* = −0.18, *p* = 0.011) were inversely associated. All predictors demonstrated acceptable multicollinearity (VIFs < 2), reinforcing the robustness of the model.

**TABLE 2:** Multiple linear regression predicting sway velocity (postural stability).

Predictor	B (unstandardized)	95% CI for B	SE	β (standardized)	t	*P*-value	VIF
PPT	0.47	0.24 to 0.70	0.12	0.31	3.92	0.0	1.42
NPRS	−0.36	−0.54 to −0.18	0.09	−0.28	−4.0	0.0	1.67
PCS	−0.29	−0.45 to −0.13	0.08	−0.25	−3.63	0.001	1.75
Mini-Cog	0.33	0.11 to 0.55	0.11	0.27	3.0	0.003	1.32
GDS	−0.18	−0.32 to −0.04	0.07	−0.21	−2.57	0.011	1.51

Dependent variable: sway velocity. Model summary: R^2^ = 0.48, F(5, 130) = 24.15, *p* < 0.001. PPT, pressure pain threshold; NPRS, Numeric Pain Rating Scale; Mini-Cog, mini-cognitive assessment instrument; GDS, geriatric; R^2^, coefficient of determination; F, F-statistic; B, unstandardized coefficient; β, standardized coefficient.

**FIGURE 4:**
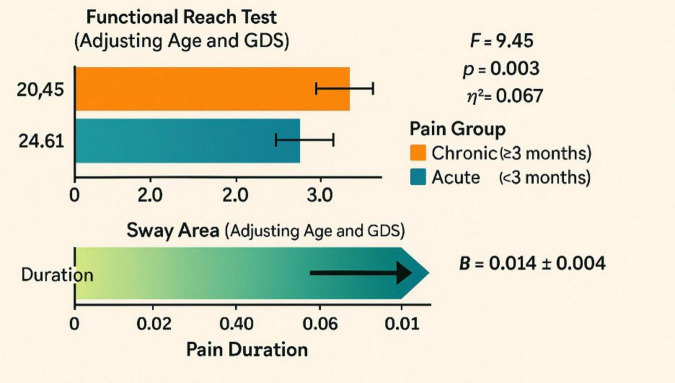
Forest plot of regression coefficients for predictors of sway velocity in older adults.

After adjusting for age and depressive symptoms, pain chronicity was found to significantly influence both functional reach and balance outcomes in older adults (Figure 4 and Table 3). Individuals with chronic pain (≥3 months) demonstrated a markedly reduced adjusted mean reach (20.45 ± 0.82 cm) compared to those with acute pain (<3 months: 24.61 ± 0.91 cm), with this group difference reaching statistical significance (*F* = 9.45, *p* = 0.003, η^2^ = 0.067). Additionally, longer pain duration was positively associated with greater sway area (*B* = 0.014 ± 0.004), indicating increased postural instability, and this relationship remained significant after adjusting for age and GDS scores, which were included as covariates based on prior evidence and preliminary correlation patterns. These findings highlight the detrimental impact of prolonged pain exposure on both functional mobility and balance control in the aging population.

**TABLE 3:** Effect of pain chronicity on functional reach and balance outcomes.

Outcome	Predictor/group	Adjusted mean (±SE)/B (±SE)	F	*p*	η^2^ (effect size)
FRT	Pain group (chronic ≥ 3 months vs. Acute < 3 months) ANCOVA adjusted for age, GDS	20.45 ± 0.82 (chronic) vs. 24.61 ± 0.91 (acute)	9.45	0.003	0.067
Sway area	Pain duration (months, continuous) Linear regression adjusted for age, GDS	*B* = 0.014 ± 0.004	11.78	0.001	0.083

FRT, functional reach test; SE, standard error; B, unstandardized coefficient; F, F-statistic; p, *p*-value; η^2^, eta squared (effect size); GDS, Geriatric Depression Scale.

## 4 Discussion

This study aimed to investigate the association between pain mechanisms–specifically pain sensitivity, intensity, and catastrophizing–and postural stability in older adults, while also examining the impact of pain chronicity and duration on functional balance outcomes. The findings revealed significant relationships between pain-related variables and measures of postural control, suggesting that both sensory and cognitive-affective aspects of pain contribute meaningfully to balance impairments in aging populations. Notably, reduced functional mobility and increased postural instability were observed in individuals with chronic pain compared to those with acute pain, highlighting the detrimental role of prolonged pain exposure. These results underscore the multifactorial nature of balance dysfunction in older adults and emphasize the importance of addressing both physiological and psychological components of pain in fall-risk assessment and intervention strategies.

The observed associations between pain mechanisms and postural control outcomes in older adults reflect the complex interplay between sensory processing, cognitive-affective modulation of pain, and functional mobility (Ge et al., 2021). Lower pressure pain thresholds correlated with both higher pain intensity and increased pain catastrophizing, suggesting heightened nociceptive sensitivity in individuals with reduced balance performance (Weaver et al., 2022). This is further reinforced by the positive relationship between pain sensitivity and functional reach, indicating that individuals with lower mechanical pain tolerance may engage compensatory motor strategies to preserve stability (Fernández-Palacios et al., 2025). The strong intercorrelations among pain intensity, catastrophizing, and sway measures underscore the integral role of psychological pain appraisal in influencing balance control (Van Wesemael et al., 2024). The consistent relationship between longer pain duration and increased postural sway supports the notion that chronic pain may contribute to neuromuscular inefficiency, possibly through persistent alterations in central processing or proprioceptive feedback, ultimately impacting balance control (Sipko et al., 2021). These findings align with existing literature that links pain-related cognitive-emotional responses to postural performance (Sipko et al., 2021). Mofateh et al. (2024) reported that higher levels of pain catastrophizing were associated with poorer dynamic balance in older adults. Similarly, Alshahrani et al. (2025) found that older individuals with chronic musculoskeletal pain exhibited increased sway and reduced functional reach, consistent with the present study’s associations between pain duration and sway area. Moreover, Sung and Lee (2025) emphasized the role of chronic pain in mobility limitation and fall risk, suggesting a mechanistic overlap between pain processing and postural regulation. The present data reinforce the cumulative evidence that both the intensity and chronicity of pain–as well as how pain is cognitively interpreted–are critically associated with balance deficits in aging populations (Dagnino and Campos, 2022).

The regression analysis revealed that multiple pain-related and psychosocial factors independently influenced sway velocity, underscoring the multidimensional nature of postural stability in older adults (Pourahmadi et al., 2023). Among these, pressure pain threshold emerged as the most significant positive predictor, indicating that individuals with heightened mechanical pain sensitivity exhibited greater instability (Vervullens et al., 2022). Conversely, pain intensity and pain catastrophizing were associated with reduced sway velocity, which may appear counterintuitive given their typical link to impaired postural control. One possible explanation is that individuals with heightened pain-related fear or cognitive distress may adopt overly cautious, rigid postural strategies–reducing sway velocity as a compensatory behavior to minimize perceived risk of instability. This phenomenon has been observed in populations exhibiting protective movement patterns in response to pain-related threat (Moustafa et al., 2025). Furthermore, cognitive function positively influenced postural control, while depressive symptoms were inversely related to stability, reflecting the broader neurocognitive and affective context of balance impairments in aging (Divandari et al., 2023). The absence of multicollinearity supports the independent contribution of these factors and affirms the robustness of the model.

These results align with earlier research demonstrating similar relationships between pain mechanisms and balance. Sarvari et al. (2024) highlighted that both pain intensity and catastrophizing significantly impacted postural control metrics in older adults. Likewise, Knox et al. (2021) reported that chronic pain was linked to impaired mobility and increased fall risk, while Peterson et al. (2023) found that cognitive performance moderated the impact of pain on physical function. Together, these studies corroborate the present findings and reinforce the importance of addressing both sensory and cognitive-emotional dimensions of pain in managing balance dysfunction among older adults (Imdad et al., 2025; Merchant et al., 2021).

### 4.1 Clinical significance

This study provides clinically relevant evidence that both physiological and psychological dimensions of pain–including pressure pain threshold, pain intensity, catastrophizing, and chronicity–are significantly associated with postural stability in older adults (Sarvari et al., 2024). By identifying sway velocity as a sensitive indicator of balance impairment, the findings underscore the importance of integrating quantitative sensory testing and pain cognition assessments into geriatric balance evaluations (Weaver et al., 2022). The demonstrated influence of cognitive status and mood on sway performance further supports the need for interdisciplinary interventions targeting not only physical rehabilitation but also cognitive and emotional health. These results emphasize that comprehensive pain assessment is critical for fall-risk stratification and the development of personalized, multimodal management strategies in older adults with persistent pain.

### 4.2 Limitations and areas for future research

The cross-sectional design permits identification of associations but precludes causal inference, leaving the directionality between pain and balance uncertain. Pain may contribute to postural instability, while instability or fear of falling may, in turn, intensify pain perception and maladaptive cognitions. Longitudinal studies are needed to disentangle these bidirectional effects and establish causality. Pressure pain threshold was measured at the upper trapezius due to its accessibility, reliability in older adults, and everyday use in quantitative sensory testing; however, this site does not capture localized sensitivity in common pain regions such as the knee or lower back. Multi-site PPT assessment in future research would enhance anatomical relevance and individual pain profiling. Additional limitations include reliance on self-reported measures of pain duration and depressive symptoms, recruitment of relatively high-functioning community-dwelling participants that may limit generalizability, and the absence of control for potential confounders such as medication use, physical activity, and comorbidities. Future studies should adopt longitudinal designs, incorporate neurophysiological measures, and evaluate multimodal interventions addressing both sensory and cognitive-emotional dimensions of pain to clarify mechanisms and improve balance outcomes.

## 5 Conclusion

This study demonstrates that both sensory and cognitive-affective aspects of pain, along with chronic pain duration, are independently associated with reduced postural stability in older adults. Sway velocity emerged as a robust balance outcome, with strong predictive contributions from pain sensitivity, intensity, catastrophizing, cognitive function, and depressive symptoms. Furthermore, individuals with chronic pain exhibited diminished functional reach and increased sway area, even after adjusting for age and mood. These findings highlight the multifactorial influence of pain on postural control and support the integration of multidimensional pain assessments in clinical balance evaluations for older adults.

## Data Availability

The datasets presented in this study can be found in online repositories. The names of the repository/repositories and accession number(s) can be found in the article/Supplementary material.
